# Effect of time-of-day on human dynamic thermal perception

**DOI:** 10.1038/s41598-023-29615-8

**Published:** 2023-02-09

**Authors:** Marika Vellei, Ilaria Pigliautile, Anna Laura Pisello

**Affiliations:** 1 Laboratory of Engineering Sciences for the Environment LaSIE (UMR CNRS 7356), La Rochelle University, La Rochelle, France; 2 Indoor Environmental Quality Laboratory, School of Architecture, Design and Planning, The University of Sydney, Camperdown, Australia; 3 Department of Engineering, University of Perugia, Perugia, Italy; 4 CIRIAF – Interuniversity Research Center on Pollution and Environment Mauro Felli – University of Perugia, Perugia, Italy

**Keywords:** Engineering, Health occupations

## Abstract

Implementing heating and cooling set-point temperature modulations in buildings can promote energy savings and boost energy flexibility. However, time and time-of-day requirements in current indoor climate regulations are either overly simplified or ignored completely. A better understanding of how human thermal responses vary throughout the day is useful to effectively design and operate energy-flexible buildings. To date, only a handful of studies have looked at diurnal changes in thermal perception and mostly near steady-state neutrality without controlling for light exposure. This is the first experimental investigation aimed at understanding how the time of the day influences physiological and subjective human sensory responses to a localized dynamic thermal stimulus under constant light rich in long wavelengths (red). Results indicated that humans responded physiologically differently depending on the time of the day with a higher rate of change in the skin temperature in the evening compared with the afternoon. Furthermore, the increase of thermal sensation during the warming skin temperature transients was found to be greater in the evening. No differences were observed under steady-state thermal conditions. This evidence suggests that accounting for the time of the day is important when dynamically operating buildings, such as during demand-response programs.

## Introduction

### Problem statement

The implementation of time-varying indoor set-point temperatures can contribute to reducing buildings’ energy consumption and promoting their energy flexibility^1,2^. Heating and cooling set-point temperature modulations can also be useful for enhancing building occupants’ comfort^3–8^, well-being^9^, and health^10–12^. However, thermal comfort has been mostly studied under steady-state conditions and near neutrality^8,13^; the effect of the time-of-day on human thermal perception has been even less often addressed^14^. As a consequence, time and time-of-day requirements in current indoor climate regulations are either over simplistic or not taken into account at all^15,16^. The supposition that thermal comfort does not depend on the time-of-day is primarily based on experimental studies conducted in the 1970s and 1980s^17–22^. More recent evidence suggests that diurnal variations in human thermal perception exist^23,24^. These observations highlight the importance of re-evaluating and re-quantifying the impact played by the time-of-day factor on human thermal comfort responses, especially under the dynamic conditions typically encountered during demand-management electricity programs, such as demand-response (DR) events. If diurnal variations in human thermal perception were confirmed, the way we currently operate buildings could be significantly modified.

### State-of-the-art

**Figure 1:**
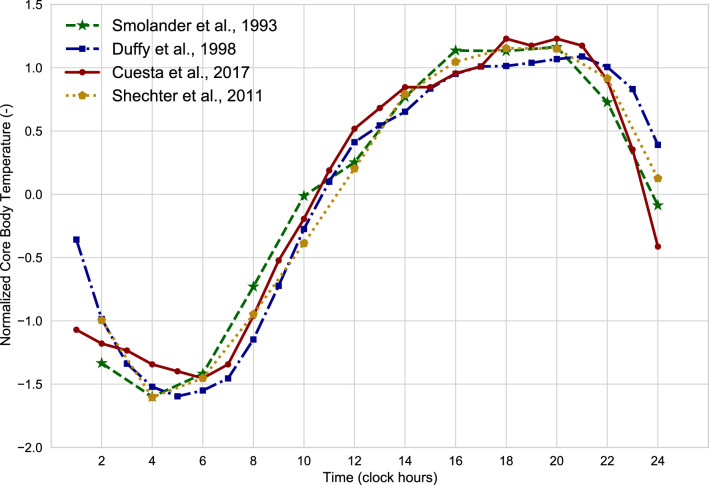
Normalized (Z-score) core body temperatures as a function of the time of day measured in controlled but not constant routine (i.e. sleep is allowed) experimental conditions. Data from Smolander et al.^30^, Duffy et al.^35^, Cuesta et al.^29^, and Shechter et al.^27^.

The lowering of the core body temperature at night is a well-known physiological phenomenon documented since 1842^25^. The extent of this change varies among individuals but it is usually around 1 °C. The nadir in core body temperature occurs during sleep time at about 04:00, see Fig. 1. Distal skin temperature (i.e. the temperature of hands and feet) also varies along the day but with inverted and higher amplitude compared to the core body temperature rhythm^26–29^. The nadir of the core body temperature is preceded by the peak in distal skin temperature by an average of 4 h^27,29,30^. These diurnal rhythms in core body and skin temperatures are due to the circadian variation of the balance between body heat generation and dissipation. Concerning heat production, the basal metabolic rate (i.e. the fasted and resting metabolic rate) is highest during the late afternoon and lowest during the late night^31^. Regarding heat loss, core body temperature is defended at a lower level during the night due to the modification of the thresholds regulating the autonomic thermoregulatory responses of vasodilation and sweating^30,32,33^. These diurnal variations are coordinated by the circadian system which makes it possible for humans to synchronize their physiological functions and behaviours to the 24-h solar day by using signals from the environment, such as light exposure, activity, body position, environmental temperature, and sleep. The circadian system consists of a central circadian clock located in the hypothalamus and peripheral clocks present in every cell of the body^34^. The period of the central clock is slightly longer than 24 h and, hence, this diurnal rhythm is referred to as circadian, i.e. *“circa diem”* or *“about a day”*.

Despite variations in core body and distal skin temperatures and autonomic thermoregulatory responses across the day being well-identified, only a few studies have examined diurnal changes in thermal perception and/or behavioural thermoregulatory responses. In a previous review^14^, we identified only 21 experimental and observational thermal comfort studies including the “time-of-day” variable^17–24,36–49^. We could observe a preference of building occupants for higher temperatures in the late afternoon (17:00 to 18:00) at a time when the core body temperature is about to reach its peak^23,24,38,42^. However, the results from at least four other studies^17–22^ were found to contradict these findings. The review outlined some possible reasons for the lack of concordance between studies, including a potential major bias associated with the intensity and spectrum of the experimental lighting conditions. Daylight is the primary synchronizer of the central circadian clock to the 24-h solar day and its effect on the circadian rhythm depends on the time of day at which light exposure occurs^50^. In the late evening and early night, bright light, and in particular short-wavelength (blue) radiation, suppresses the release of nocturnal melatonin and slows down the increase in distal skin temperature and the associated decline in core body temperature^51^, which induces phase delays of the circadian rhythm^52^. The maximum melatonin-suppressing effect is achieved at the shortest wavelengths (i.e. 424 nm, violet)^53^. In the late night and early morning, bright light exposure anticipates the increase in core body temperature and the decline in distal skin temperature, which results in phase advances^54^. Despite the well-documented role of light in synchronizing the human circadian rhythmicity, only three out of the 15 reviewed experimental studies reported the characteristics of the lighting conditions and only in terms of intensity (lux)^17,21,42,43^. None of the six reviewed observational studies conducted any light measurements. Furthermore, the majority of the studies were conducted in thermoneutral steady-state conditions. The effect of the time-of-day on thermal comfort may be more significant under dynamic conditions and at the boundaries of the thermoneutral zone while being insignificant near the centre of thermoneutrality. Thus, the circadian effect on human thermal perception may be masked by the fact that previous studies were mostly conducted in steady-state thermoneutral conditions.

### Research aim

The primary research aim of this study is to understand whether the time of the day (afternoon and evening) influences physiological (skin temperature) and subjective (thermal sensation and thermal pleasure) human thermal responses to a fan-induced dynamic localized thermal stimulus. As secondary objectives, this study also aims to investigate sex differences and the effect of thermal alliesthesia on human dynamic thermal perception. The experiment is performed under constant light condition rich in long wavelengths (red) that is not capable of suppressing the decline of core body temperature in the evening^55–57^. We set the following a priori null hypotheses:

1. The time of the day does not affect human dynamic thermal perception.
2. Human dynamic thermal perception does not differ between females and males.

## Methods

### Experimental procedure

The study was conducted during June and September 2021 (northern hemisphere summer) in the NEXT.ROOM, a test room developed for human comfort studies at the CIRIAF Institute, the interuniversity research centre on Pollution and Environment *“Mauro Felli”* in Perugia, Italy. The mean daily outdoor temperature was 22.8 °C during the experiments in June and 20.5 °C in September.

**Figure 2:**
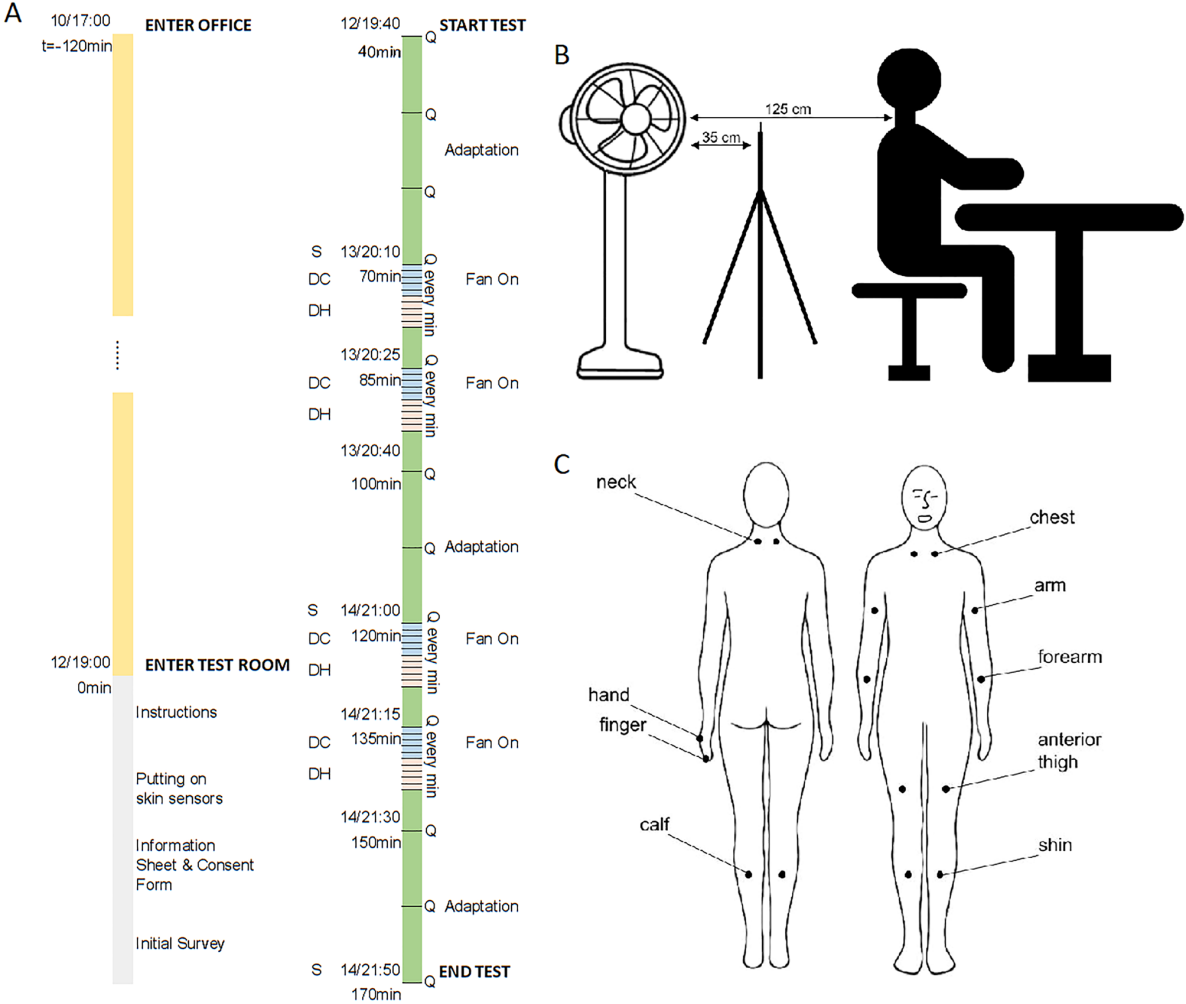
Experimental procedure (**A**), setup (**B**), and overview of the 16 skin measurement locations (**C**). “Q” indicates the times the questionnaires are filled out. “DC” and “DH” indicate the four sequences of partial-body active cooling and passive warming respectively. “S” indicates the times when steady-state thermal conditions are reached.

A fan was used to expose the participants to four sequences of partial-body cooling over 130 min under a neutral whole-body thermal condition as predicted by Fanger’s PMV model. The active cooling of the skin induced by the fan was followed by a passive re-warming towards the neutral condition. The four cycles were selected to induce thermal alliesthesia by alternating displeasure and pleasure thermal states. After the first two cycles, the body was allowed to return to a steady state for 30 min. The fan was mounted 125 cm away from the back of the neck of the participant when seated straight. The anemometer was 35 cm in front of the fan and 90 cm behind the participant’s neck. Seat height was adjusted to ensure that each participant’s neck was directly in the path of the airflow. The experimental procedure and setup are shown in Fig. 2. Both subjective questionnaires (thermal sensation and thermal pleasure) and physiological (skin temperature) measurements were collected during the exposure.

The same experimental procedure was repeated at two different times of the day (afternoon and evening, i.e. starting at 12:00 pm and 19:00). Eight participants (four males and four females) were exposed to the neutral condition starting at 12:00 pm, and six other participants (three males and three females) at 19:00. The light condition was identical at the different times of the day. We used a polychromatic light with an illuminance of about 200 lx, a Dominant Wavelength (DWl) of about 580 nm and a Correlated Colour Temperature (CCT) of about 3400 K (red colour) as measured at the level of the eye of the subject in the main direction of gaze. We selected a light condition with long wavelengths to limit the effect of short-wavelength radiation on the suppression of the decline of the core body temperature in the evening^55–57^. The most often used vertical illuminance at the eye was equal to 500 lx which can be considered a reasonable level for office tasks. The horizontal and vertical spectral irradiances are reported respectively in Supplementary Table S1 and S2 online.

One participant at a time took part in the experiment and only at one time of the day (i.e. between-subjects experimental design). On the day of the experiment, the participant arrived at least 2 h before the beginning of the study and was asked to remain in a normal office for metabolic adaptation purposes before being transferred to the test room. The office was monitored in terms of air temperature but the environmental conditions were not controlled. For the tests starting at 12:00 pm, participants were advised not to have any food from 10:00. For those starting at 19:00, they were advised not to have any food from 17:00. An energy bar (about 200 kcal) was provided at the beginning of the test. Bottled water was also made available. For the first 70 min of the exposure, the thermal conditions were kept constant to allow the occupant to reach steady-state thermal conditions before the start of the fan-induced active cooling and passive warming transients. During the first 30 min in the test room, each participant filled out a questionnaire about personal information and was briefed verbally about the study’s requirements and allowed to ask any questions but without detailing the thermal conditions that he/she was going to experience, thus following a single-blind procedure. This time was also used to put the sensors on the participant’s skin. The first thermal perception questionnaire was completed 40 min after entering the test room and this was considered the actual start of the test. The principal investigator of the study (i.e. the first author) was continuously present in the test room during all experimental sessions to operate the fan and make sure the participants were answering the questionnaires at the right times.

### Experimental platform

The experiment took place in the NEXT.ROOM, a test room developed for human comfort studies at the CIRIAF Institute in Perugia, Italy. The test room is located within the laboratories in the basement of the main building of the Institute and presents an internal volume of 4 × 4 × 2.7 m. It includes a unique window, aligned to the building openings to provide a view of the outdoors from inside the room. During the experiments, the window was closed by a 0.05 m thick opaque panel of polystyrene to avoid daylight access to the space and limit heat fluxes through the envelope. The lighting scenario as described in the previous section was realized by controlling the six luminaires at the ceiling of the NEXT.ROOM. The internal thermal conditions were achieved thanks to the control of the radiant surfaces (capillary tube system fit within the pre-cut guides of the polystyrene panels, connected to a reversible air-to-water heat pump) and a heat-recovery HVAC system. The conditioned supply air was distributed from four inlet nozzles located on one side of the room (two on the bottom of the wall and two on the ceiling). On the opposite side of the test room’s ceiling, two air extractors took away the exhaust air. Detailed technical descriptions of the NEXT.ROOM (including several figures illustrating the layout of the test room and the relevant HVAC specifications) are provided in Vittori et al*.*^58^.

### Participants

Fourteen adults (seven males and seven females) participated in the experiment. The participants were recruited by word of mouth, mostly within the University of Perugia. Only participants between 20 and 40 years old were included in the study to avoid any confounding effect of the age variable. They were asked to wear similar clothing outfits, consisting of short trousers or a skirt, a shirt with short sleeves, ankle-length socks and shoes with total clothing insulation estimated to be about 0.6 clo (including the insulation of the office chair), based on the tabulated clo values given in ANSI/ASHRAE Standard 55^15^.

During the experiments, they were allowed to perform office tasks (reading or studying, using their mobile phones, working at the computer or performing other non-physical activities) but were not permitted to move around the room. Their metabolic rate was estimated to be constant and equal to approximately 1 met. For at least 24 h before the experiment, they were requested to:

- Avoid heavy exercise,
- Avoid alcoholic or stimulating drinks,
- Avoid eating large meals,
- Maintain a regular sleep schedule (do not stay overnight the night before).

All the participants were paid for their participation. The anthropometric characteristics of the participants are reported in Table 1. Three out of seven female participants were taking oral contraceptives. None of the participants reported regular night work or a change of time zone in the two months before the study.

**Table 1:** Anthropometric characteristics (mean ± SD) of the participants.

	Afternoon		Evening	
	Male (4)	Female (4)	Male (3)	Female (3)
$\mathrm{Age}$(years)	32 ± 4	31 ± 6	30 ± 1	30 ± 3
$\mathrm{Height}$(m)	1.80 ± 0.08	1.60 ± 0.03	1.79 ± 0.05	1.65 ± 0.06
$\mathrm{Weight}$(kg)	73.0 ± 3.6	54.5 ± 7.8	73.3 ± 11.7	54.7 ± 4.2
$\mathrm{BMI}$(kg/m^2^)	22.7 ± 2.4	21.2 ± 3.1	22.7 ± 2.7	20.1 ± 1.9

### Measurements

#### Environmental

Air temperature ${T}_{a}$ (RTD, Metron manufacturer, accuracy ± 0.1 °C), globe temperature ${T}_{g}$ (RTD, accuracy ± 0.1 °C), relative humidity $RH$ (Delta OHM manufacturer, model HD20001.1, accuracy ± 1.5%), air velocity ${V}_{a}$ (hot wire anemometer, Delta OHM manufacturer, model HD4V3TS(4), accuracy ± 0.2 + 3%), surface temperature (type T, Metron manufacturer, accuracy ± 0.1 °C), carbon dioxide ${CO}_{2}$ (E + E Elektronik manufacturer, model EE80-2CTF3/T04, accuracy ± 50 ppm + 2%), and illuminance $ILL$ (Delta Ohm manufacturer, model HD2021T, accuracy < 5%) were recorded. The lighting characterization was carried out through a JETI Specbos 1211UV spectroradiometer (wavelength range 350 ÷ 1000 nm). The air temperature was monitored at 0.1, 0.6, and 1.1 m height on one side of the participant at a distance of about 40 cm^59^, and an average air temperature was calculated over the three values. The mean radiant temperature was computed from the globe temperature using the function *psychrometrics.t_mrt* from the *pythermalcomfort* python package^60^. Fanger’s $PMV$ index was also computed using this package.

#### Physiological

Skin temperature was measured in 16 areas of the body: neck (${T}_{skin, neck}$), chest (${T}_{skin, chest}$), arm (${T}_{skin, arm}$), forearm (${T}_{skin, forearm}$), anterior thigh (${T}_{skin, ant \,thigh}$), calf (${T}_{skin, calf}$), and shin (${T}_{skin, shin}$) at both the left and right sides of the participant plus hand (${T}_{skin, hand}$) and finger (${T}_{skin, finger}$) at the left side. See Fig. 2 for the exact placement of the thermocouples. The finger thermocouple was on the back of the 4th finger, close to the fingernail. All body-part skin measurements, except hand and finger, were calculated as the mean of the left and right sides of the body. The distal skin temperature (${T}_{skin, distal}$) was calculated as the mean of the finger and hand skin temperatures. Skin temperatures were measured every 30 s using contact thermometry which consists of temperature sensors positioned in direct contact with the skin surface and, therefore, relies on conductive heat exchange between skin and sensor. We used thermocouples of type T (± 0.2 °C accuracy, TCSA manufacturer), also known as copper–constantan thermocouples, with a diameter of 0.2 mm. The thermocouples were connected to a multichannel data acquisition system (Campbell CR1000 data logger + AM16/32B multiplexer). Before taking the measurements, the thermocouple system was calibrated for the temperature range of 20 to 50 °C. These thin thermocouples were fixed onto the skin with a breathable medical tape ensuring good contact and a rapid response time (< 10 s), which is necessary for the dynamic conditions being studied.

Mean skin temperature (${T}_{skin \,mean}$) was estimated using the formula proposed by Ramanathan^61^ based on four weighted body locations:

1 $${T}_{skin \,mean}=0.3*{T}_{skin,chest}+{0.3*T}_{skin,arm}+0.2*{T}_{skin,calf}+0.2*{T}_{shin, ant \,thigh}.$$

For each skin temperature location and at each minute, we calculated the rate of change of the skin temperature as the first-order derivative with respect to time ($\frac{{\partial T}_{skin}}{\partial t}=\frac{{T}_{skin,t}{-T}_{skin,t-1}}{\partial t}$).

#### Subjective

The participants filled in a questionnaire describing their whole-body thermal sensation and thermal pleasure at 10 min intervals, except during the partial-body cooling and warming periods where the questions were asked every minute. The exact timing of the questionnaires is shown in Fig. 2. Thermal sensation is the descriptive or objective dimension of thermal perception, while the affective or hedonic component of thermal perception can be assessed in terms of thermal pleasure^62^. Thermal pleasure (and not thermal sensation) is important for activating purposive thermoregulatory behaviours which have been described as *“an attempt to avoid what humans call thermal discomfort or displeasure and to obtain thermal pleasure”*^63,64^. The Thermal Sensation Vote (TSV) was asked on the classical ASHRAE 7-point thermal sensation scale ranging from *“Cold”* (− 3) to *“Hot”* (+ 3)^15^. The Thermal Pleasure Vote (TPV) was also asked on a 7-point scale: *“Very pleasant”* (+ 3), *“Pleasant”* (+ 2), *“Slightly pleasant”* (+ 1), *“Indifferent”* (0), *“Slightly unpleasant”* (− 1), *“Unpleasant”* (− 2), and *“Very unpleasant”* (− 3). The questions were either in English or Italian depending on the language preferred by the participants and were presented through an Internet browser. Only discrete votes were allowed. The two questions translated into Italian are reported in the Supplementary Note.

### Ethics

The research protocol was approved by the Ethics Committee at the University of Tours and Poitiers in France (Protocol No. CER-TP 2021-06-02) and adhered to the principles of the Declaration of Helsinki. Each participant was provided with written instructions and an information sheet and gave written informed consent to participate in the study.

### Statistical analysis

Statistical differences between the means of two independent samples (from two groups) at each time point are calculated with the non-parametric *Mann–Whitney U* test. The common language effect size (*CLES*) is used as a measure of effect size and indicates the probability that a random value or score from one group will be greater than a random value or score from the other^65^. A Mixed-effects Linear Model (MLM) with possible two-way interactions is employed to model the thermal sensation vote as a function of the skin temperature, the rate of change of skin temperature, sex, and the time of day that are included as fixed effects. The participants are treated as random factors (random intercept model) due to the longitudinal nature of the collected time-series data. The maximum likelihood is the chosen estimation method for the parameters in the MLM model. A *“top-down”* modelling strategy is used, starting with the maximum model followed by a stepwise backward elimination procedure with only significant covariates kept in the model at the end of the procedure. The open-source Python packages *pingouin* and *statsmodels* are used for the statistical analyses. Differences at *p* ≤ 0.05 are considered statistically significant.

The sample size of fourteen participants for the testing of the first null hypothesis was chosen based on a prospective power analysis calculation. None of the papers identified in the literature review reported effect size so the rationale for the selection of the effect size was to have a study capable of detecting a shift effect of 1 vote on the Likert scale. With the power levels set to 0.80 (i.e. a probability of making a Type II error equal to 20%), the alpha levels set at *p* < 0.05 (i.e. a probability of making a Type I error equal to 5%), for a nondirectional (two-tailed) *Mann–Whitney U* test, the required sample size corresponded to fourteen participants^66^.

## Results

### Overview

#### Environmental

The mean measured environmental conditions during the four experimental exposures are reported in Table 2. The mean vertical gradient between the air temperature measured at 0.1 and 1.1 m was equal to 0.3 °C so the stratification was not important. Throughout the experiment, the air velocity was kept under 0.1 m/s, except for the time steps when the fan was used. During the first two fan series, the air velocity was equal to 2.7 m/s, while during the last two fan series was equal to 3 m/s.

**Table 2:** Measured environmental conditions (mean ± SD).

	${T}_{a}$(°C)	${T}_{r}$(°C)	$RH$(%)	$PMV$(-)	${CO}_{2}$ (ppm)
AFTERNOON	27.0 ± 0.3	27.6 ± 0.5	41.4 ± 4.8	0.35 ± 0.2	550 ± 16
EVENING	26.9 ± 0.1	27.5 ± 0.1	42.0 ± 4.1	0.34 ± 0.1	559 ± 16

#### Physiological

**Figure 3:**
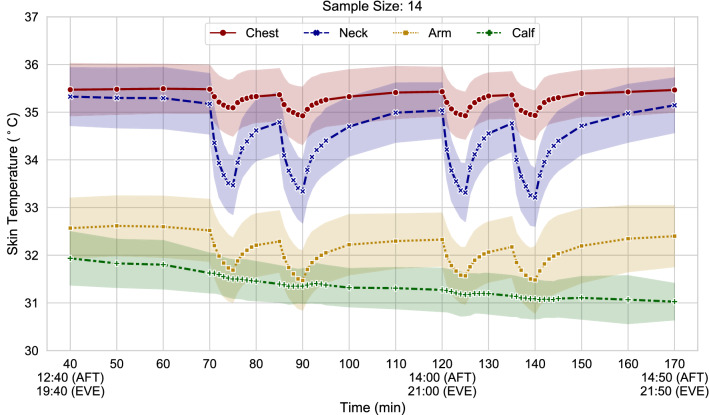
Skin temperature over the course of the experiment at 4 different locations (chest, neck, arm, and calf). Shaded bands represent one standard deviation.

**Figure 4:**
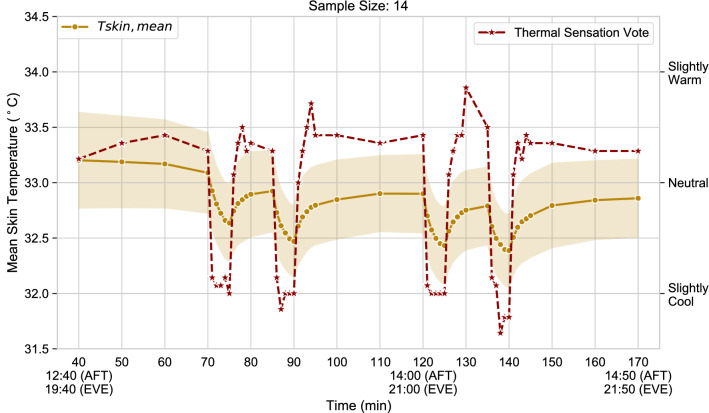
Mean skin temperature (left) and thermal sensation vote (right) over the course of the experiment. Shaded bands represent one standard deviation.

Figure 3 gives an overview of the chest, neck, arm, and calf skin temperatures throughout the experiment. From Fig. 3 we can see that the local skin temperatures of cold-affected body parts (neck and arm) could not rewarm completely during the recovery period and return to baseline levels. We refer to this phenomenon as thermal habituation and define it as a short-term (i.e., of the order of minutes or hours) adaptive process that modifies the body's response after non-neutral thermal exposures^8^. This phenomenon appears to occur at a central level, rather than being related to the dynamic activity of sensory neurons as the dynamic thermal overshoot phenomena (see next section “Subjective”)^67^. Thermal habituation has been suggested to be related to blood vessels needing time to recover after cool exposures, probably to avoid excessive fluctuations^68–71^. From Fig. 3 we can also observe that the calf skin temperature, and, in general, the lower body skin temperatures decreased with time. Despite the mean skin temperature decreasing over time (see also Fig. 4), the participants’ mean thermal sensation was not decreasing after the fan exposures. This is in line with previous observations showing that thermal habituation causes the thermal sensation to shift in the opposite direction to the preceding thermal sensation, i.e*.* nudged towards a warmer sensation after cool exposures^70,71^.

#### Subjective

The term *“thermal overshoot”* refers to the anticipatory and overshooting sensory behaviour observed under dynamic thermal conditions^72,73^. This phenomenon primarily depends on the ability of sensory neurons to detect the rate of change of the skin temperature and to send this information to the brain through spiking of their firing rate^74,75^. The thermal overshoot phenomenon is evident in Fig. 4 which shows the thermal sensation against the mean skin temperature recorded throughout the experiment. During the skin cooling transients, the thermal sensation could anticipate the body temperature changes and predict the final steady-state sensory response. While during the passive skin warming transients, the thermal sensation could also initially exaggerate the final steady-state sensory response since some sensory peaks can be noticed.

### Time-of-day

**Figure 5:**
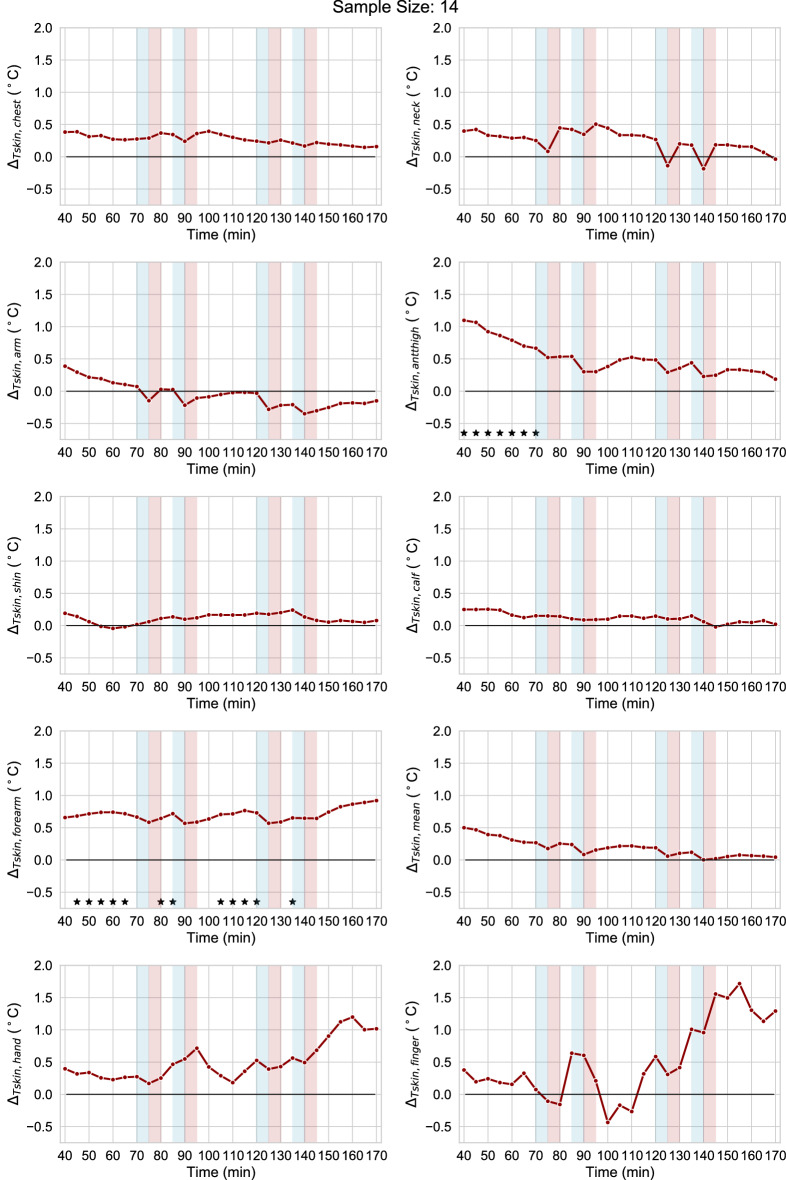
Mean difference (Δ) between the evening and afternoon values of the skin temperature at different locations. Asterisks indicate statistically significant differences.

To display time-of-day differences throughout the tests, we first derive Fig. 5 by grouping the resampled 5 min skin temperature data at each location based on the time of day, i.e., evening (tests starting at 19:00) and afternoon (tests starting at 12:00 pm). We then calculate the mean difference between the evening and afternoon values. Asterisks in Fig. 5 indicate statistically significant time-of-day differences. The results of the statistical tests are reported in Supplementary Table S3 online. Apart from the shin and the calf skin temperatures, all the other skin temperatures were slightly higher in the evening than in the afternoon during the adaptation time, i.e. up to t = 70 min from the beginning of the test. This was especially evident for the forearm and anterior thigh (statistically significant differences) and was probably due to the participants being exposed to higher air temperatures during the time spent in the office before entering the test room in the evening. The mean air temperature in the office before the evening tests was equal to 27.5 °C, while it was 25 °C before the afternoon tests. After the 70 min adaptation time, only the forearm skin temperature was statistically significantly higher in the evening compared to the afternoon. While not statistically significant, the difference between the evening and afternoon values of the hand and finger skin temperatures had an increasing trend in the last hour. It is to be highlighted that participants’ hand and finger skin temperatures were decreasing throughout the experiments but their decrease was much less pronounced in the late evening which explained the observed higher distal skin temperature in the late evening compared to the late afternoon. This is an indication of circadian rhythmicity and is in line with previous investigations showing that the maximal value of distal skin temperature is reached in the early night and precedes the nadir of the core body temperature by an average of four hours^27,29,30^. Forearm skin temperatures followed a similar trend to the distal skin temperature; this is probably because the thermocouples were attached in the vicinity of the wrist whose skin temperature has been shown to follow similar patterns to the distal skin temperatures^76^.

**Figure 6:**
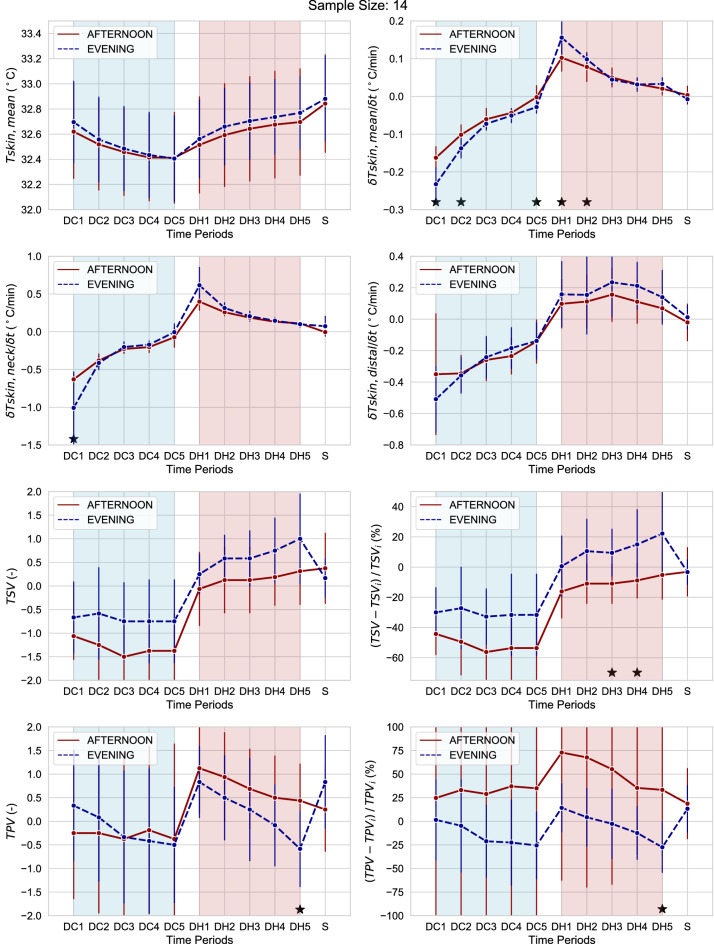
Skin temperature at various locations, subjective thermal sensation and thermal pleasure vote and their rate of change in the afternoon and evening averaged over the course of the last two partial-body cooling and warming transients. Thus, only data collected after 120 min from the beginning of the test are reported. Asterisks indicate statistically significant differences. Error bars represent one standard deviation.

To analyse the dynamic sensory responses as a function of the time of the day by limiting at a maximum the influence of the initial thermal states, we decided to only consider the data collected after 120 min from the start of the test. Thus, Fig. 6 is derived by averaging the 1 min values of skin temperatures and subjective votes only over the two last sequences of partial-body cooling and warming. To further eliminate any effect of the initial conditions we calculated two additional variables which represent the percentage change of the thermal sensation and thermal pleasure vote with respect to the initial steady-state value at 120 min, i.e. $(TSV-TSVi)/TSVi$ and $(TPV-TPVi)/TPVi$ respectively. These two variables allow us to observe the normalized trends of the subjective votes. From Fig. 6 we can notice that the rates of change of the mean skin temperature during the cooling (DC1, DC2 and DC5) and warming (DH1 and DH2) transients were statistically significantly higher in the evening compared with the afternoon. The effect size was large and the probability that a value randomly sampled from the evening group was greater than a value randomly sampled from the afternoon group was more than 80%. No difference in mean skin temperature was observed between the afternoon and evening. We can further observe that during the warming transients DH3 and DH4 participants felt warmer in the evening compared with the afternoon and the relative increase of the thermal sensation vote was statistically significantly higher in the evening with a CLES higher than 80%. The thermal sensation and thermal pleasure votes during the steady-state conditions (S) were similar between evening and afternoon. It is to be also noted that participants felt more thermally displeased in the evening during the passive warming phase at period DH5 and the difference was statistically significant. Results of the statistical tests are reported in Tables 3, 4, 5 and 6.

**Table 3:** Time-of-day differences in mean skin temperature and its rate of change over the course of the last two partial-body cooling and warming transients.

	${T}_{skin,mean}$						${\partial T}_{skin,mean}/\partial t$					
	Afternoon (8) (mean ± SD)		Evening (6) (mean ± SD)		p-value (*MWU)*	CLES	Afternoon (8) (mean ± SD)		Evening (6) (mean ± SD)		p-value (*MWU)*	CLES
DC1	32.62	0.35	32.7	0.3	0.75	0.44	**− 0.16**	**0.03**	**− 0.23**	**0.04**	**0.01**	**0.92**
DC2	32.52	0.34	32.56	0.31	0.85	0.46	**− 0.10**	**0.02**	**− 0.14**	**0.02**	**0.02**	**0.88**
DC3	32.46	0.32	32.49	0.31	0.95	0.48	− 0.06	0.03	**− **0.07	0.01	0.41	0.65
DC4	32.41	0.32	32.43	0.31	0.85	0.46	− 0.04	0.01	− 0.05	0.02	0.28	0.69
DC5	32.41	0.34	32.41	0.31	1.00	0.50	**0.00**	**0.03**	**− 0.03**	**0.01**	**0.04**	**0.83**
DH1	32.51	0.36	32.56	0.28	0.85	0.46	**0.10**	**0.03**	**0.16**	**0.04**	**0.02**	**0.12**
DH2	32.59	0.38	32.66	0.28	0.75	0.44	**0.08**	**0.04**	**0.10**	**0.01**	**0.04**	**0.17**
DH3	32.64	0.39	32.70	0.28	0.75	0.44	0.05	0.02	0.04	0.01	0.95	0.52
DH4	32.68	0.40	32.74	0.27	0.75	0.44	0.03	0.01	0.03	0.02	1.00	0.50
DH5	32.70	0.40	32.77	0.27	0.75	0.44	0.02	0.02	0.03	0.01	0.14	0.25
S	32.84	0.36	32.88	0.31	0.85	0.46	0.00	0.02	− 0.01	0.01	0.57	0.60

Significant values are in bold.

**Table 4:** Time-of-day differences in the rate of change of the neck and distal skin temperature over the course of the last two partial-body cooling and warming transients.

	${\partial T}_{skin,neck}/\partial t$						${\partial T}_{skin,distal}/\partial t$					
	Afternoon (8) (mean ± SD)		Evening (6) (mean ± SD)		p-value (*MWU)*	CLES	Afternoon (8) (mean ± SD)		Evening (6) (mean ± SD)		p-value (*MWU)*	CLES
DC1	**− 0.63**	**0.08**	**− 1.01**	**0.43**	**0.04**	**0.83**	− 0.35	0.36	− 0.51	0.19	0.75	0.56
DC2	− 0.38	0.08	− 0.41	0.08	0.75	0.56	− 0.34	0.11	− 0.36	0.10	0.66	0.58
DC3	− 0.23	0.06	− 0.2	0.06	0.57	0.40	− 0.26	0.12	− 0.24	0.12	0.66	0.42
DC4	− 0.2	0.07	− 0.17	0.04	0.49	0.38	− 0.23	0.11	− 0.18	0.12	0.57	0.40
DC5	− 0.07	0.12	− 0.01	0.10	0.11	0.23	− 0.14	0.13	− 0.14	0.11	0.85	0.46
DH1	0.40	0.11	0.62	0.22	0.06	0.19	0.10	0.14	0.16	0.19	0.57	0.40
DH2	0.26	0.04	0.31	0.06	0.11	0.23	0.11	0.16	0.15	0.23	0.95	0.52
DH3	0.19	0.04	0.21	0.06	0.57	0.4	0.16	0.16	0.23	0.20	0.66	0.42
DH4	0.13	0.02	0.15	0.04	0.66	0.42	0.11	0.13	0.21	0.14	0.28	0.31
DH5	0.11	0.02	0.10	0.04	0.66	0.58	0.07	0.08	0.14	0.16	0.57	0.40
S	0.00	0.04	0.07	0.12	0.23	0.29	− 0.02	0.11	0.01	0.07	0.41	0.35

Significant values are in bold.

**Table 5:** Time-of-day differences in thermal sensation vote and its relative change over the course of the last two partial-body cooling and warming transients.

	$TSV$						$(TSV-TSVi)/TSVi$					
	Afternoon (8) (mean ± SD)		Evening (6) (mean ± SD)		p-value (*MWU)*	CLES	Afternoon (8) (mean ± SD)		Evening (6) (mean ± SD)		p-value (*MWU)*	CLES
DC1	− 1.06	0.46	− 0.67	0.69	0.49	0.39	− 44.27	12.83	− 30	14.91	0.06	0.2
DC2	− 1.25	0.71	− 0.58	0.89	0.18	0.28	− 49.48	20.46	− 27.22	24.75	0.13	0.25
DC3	− 1.5	0.61	− 0.75	0.75	0.13	0.25	− 56.25	19.09	− 32.78	16.71	0.06	0.19
DC4	− 1.38	0.74	− 0.75	0.80	0.23	0.30	− 53.65	20.14	− 31.67	24.63	0.19	0.28
DC5	− 1.38	0.74	− 0.75	0.80	0.23	0.30	− 53.65	20.14	− 31.67	24.63	0.19	0.28
DH1	− 0.06	0.73	0.25	0.38	0.37	0.35	− 16.15	16.46	0.56	18.3	0.13	0.27
DH2	0.12	0.65	0.58	0.45	0.22	0.30	− 10.94	12.31	10.56	19.29	0.07	0.21
DH3	0.12	0.65	0.58	0.53	0.26	0.31	**− 10.94**	**12.31**	**9.44**	**14.33**	**0.04**	**0.17**
DH4	0.19	0.56	0.75	0.63	0.20	0.29	**− 8.85**	**10.91**	**15**	**20.97**	**0.04**	**0.17**
DH5	0.31	0.66	1.00	0.87	0.28	0.32	− 5.21	14.99	22.22	26.64	0.06	0.2
S	0.38	0.70	0.17	0.37	0.72	0.55	− 3.12	14.99	− 3.33	7.45	1.00	0.49

Significant values are in bold.

**Table 6:** Time-of-day differences in thermal sensation vote and its relative change over the course of the last two partial-body cooling and warming transients.

	$TPV$						$(TPV-TPVi)/TPVi$					
	Afternoon (8) (mean ± SD)		Evening (6) (mean ± SD)		p-value (*MWU)*	CLES	Afternoon (8) (mean ± SD)		Evening (6) (mean ± SD)		p-value (*MWU)*	CLES
DC1	− 0.25	1.30	0.33	1.07	0.38	0.35	24.79	115.7	1.53	39.06	0.70	0.43
DC2	− 0.25	1.58	0.08	1.24	0.79	0.45	33.13	148.79	− 4.72	44.73	0.75	0.44
DC3	− 0.38	1.71	−0.33	1.28	1.00	0.51	28.96	151.50	− 21.11	34.85	0.95	0.48
DC4	− 0.19	1.66	− 0.42	1.40	0.74	0.56	37.08	150.41	− 22.50	41.21	0.80	0.55
DC5	− 0.38	1.88	− 0.50	1.12	0.89	0.47	35.00	169.65	− 25.56	32.42	0.75	0.44
DH1	1.12	0.89	0.83	0.69	0.69	0.57	72.92	126.64	14.31	23.33	0.27	0.69
DH2	0.94	0.88	0.50	0.82	0.55	0.60	67.71	128.59	4.31	27.93	0.22	0.71
DH3	0.69	0.79	0.25	0.99	0.35	0.66	55.21	114.03	− 2.64	33.75	0.19	0.72
DH4	0.50	0.83	− 0.08	0.79	0.33	0.67	35.42	65.98	− 12.36	25.64	0.12	0.76
DH5	**0.44**	**0.73**	**− 0.58**	**0.73**	**0.05**	**0.82**	**33.33**	**65.22**	**− 27.50**	**24.34**	**0.02**	**0.89**
S	0.25	0.83	0.83	0.90	0.28	0.33	18.75	34.80	13.33	22.67	1.00	0.49

Significant values are in bold.

### Sex

**Figure 7:**
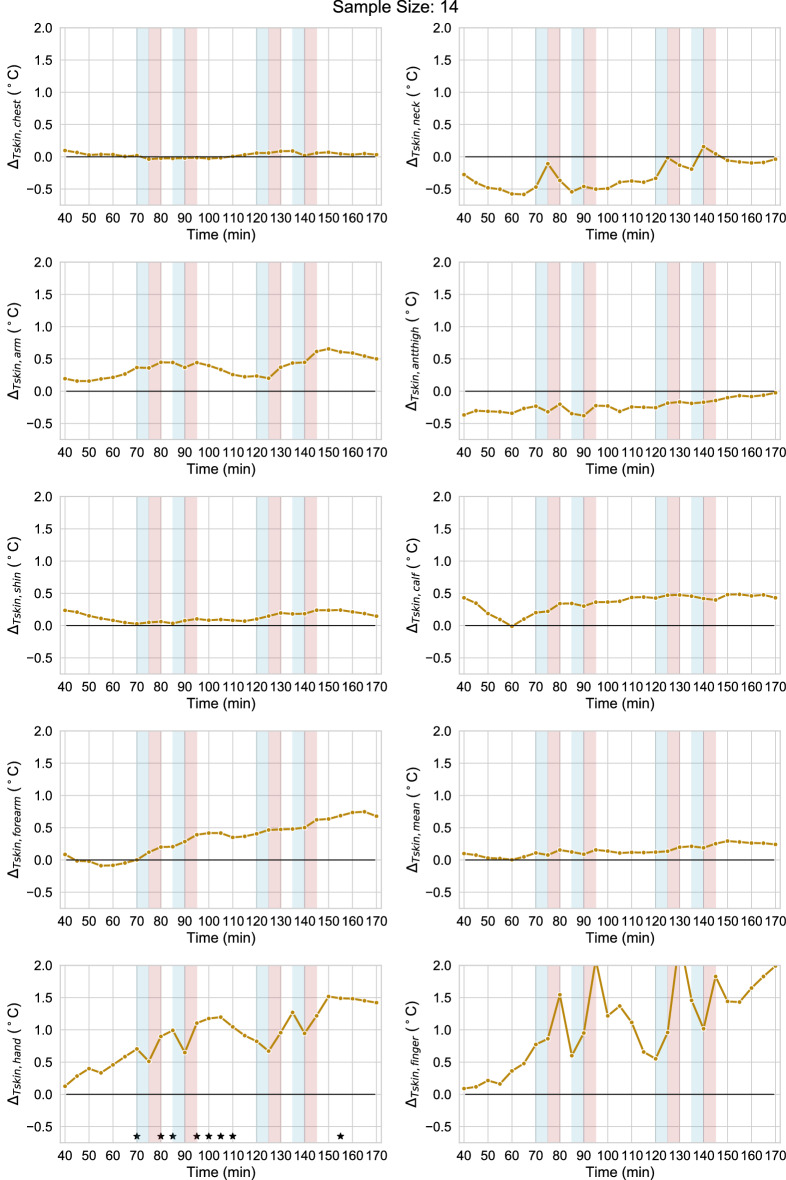
Mean difference (Δ) between male and female values of the skin temperature at different locations. Asterisks indicate statistically significant differences.

**Figure 8:**
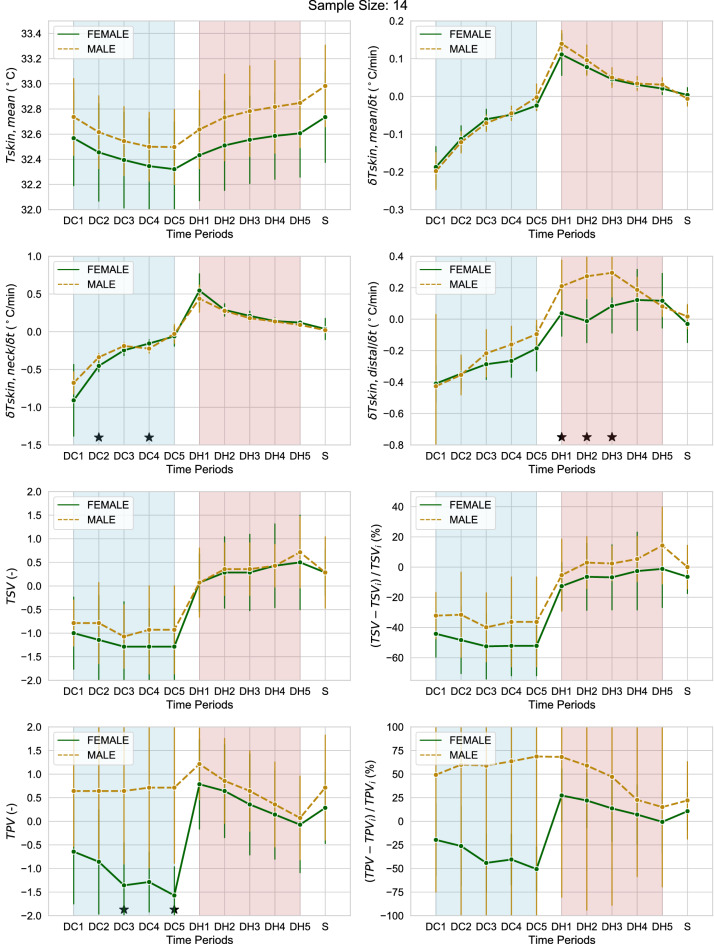
Skin temperature at various locations, subjective thermal sensation and thermal pleasure vote and their rate of change for females and males averaged over the course of the last two partial-body cooling and warming transients. Thus, only data collected after 120 min from the beginning of the test are reported. Asterisks indicate statistically significant differences. Error bars represent one standard deviation.

In this section, we compare males and females in terms of the observed skin temperatures and subjective votes. Figures 7 and 8 are derived the same as Figs. 5 and 6. The results of the statistical tests for Fig. 7 are reported in Supplementary Table S4 online. From Fig. 7 we can first observe that males had a slightly higher skin temperature than females and this is particularly evident at the lower body and distal skin locations towards the end of the exposure. This was due to the females cooling down more than males at these locations. Statistically, significant differences were only observed at the hand skin locations. A statistically significant sex difference in the neck skin temperature was also observed during the cooling transient DC2 and DC4 induced by the fan (Fig. 8). The mean decrease of neck skin temperature was faster in females than males at DC2 pointing to greater female vasoconstriction in this body part, which was the one directly in the path of the airflow. The greater decrease in neck skin temperature was accompanied by a general tendency to vasoconstriction in females with a statistically significantly greater decrease in distal skin temperature in the successive periods DH1; DH2 and DH3 and a CLES higher than 80%. Concerning the subjective votes, the lower mean skin temperature in females translated to a lower mean thermal sensation vote during cooling transients but the difference was not statistically significant. However, thermal pleasure vote was statistically significantly lower for females than males at periods DC3 and DC5. Results of the statistical tests are reported in Tables 7, 8, 9 and 10.

**Table 7:** Sex differences in mean skin temperature and its rate of change over the course of the last two partial-body cooling and warming transients.

	${T}_{skin,mean}$						${\partial T}_{skin,mean}/\partial t$					
	Female (7) (mean ± SD)		Male (7) (mean ± SD)		p-value (*MWU)*	CLES	Female (7) (mean ± SD)		Male (7) (mean ± SD)		p-value (*MWU)*	CLES
DC1	32.57	0.35	32.74	0.28	0.38	0.35	− 0.19	0.05	− 0.20	0.05	0.80	0.55
DC2	32.46	0.36	32.62	0.27	0.38	0.35	− 0.11	0.03	− 0.12	0.03	0.53	0.61
DC3	32.39	0.35	32.54	0.25	0.46	0.37	− 0.06	0.02	− 0.07	0.02	1.00	0.51
DC4	32.35	0.35	32.50	0.25	0.53	0.39	− 0.05	0.01	− 0.04	0.02	0.62	0.41
DC5	32.32	0.35	32.50	0.28	0.38	0.35	− 0.02	0.01	0.00	0.03	0.10	0.22
DH1	32.43	0.33	32.64	0.29	0.32	0.33	0.11	0.05	0.14	0.03	0.10	0.22
DH2	32.51	0.33	32.73	0.32	0.38	0.35	0.08	0.02	0.10	0.04	0.53	0.39
DH3	32.56	0.32	32.78	0.33	0.38	0.35	0.05	0.01	0.05	0.02	1.00	0.49
DH4	32.59	0.32	32.82	0.34	0.38	0.35	0.03	0.01	0.03	0.02	0.62	0.41
DH5	32.61	0.32	32.85	0.33	0.32	0.33	0.02	0.01	0.03	0.02	0.46	0.37
S	32.74	0.33	32.98	0.30	0.32	0.33	0.00	0.02	− 0.01	0.02	0.21	0.71

**Table 8:** Sex differences in the rate of change of the neck and distal skin temperature over the course of the last two partial-body cooling and warming transients.

	${\partial T}_{skin,neck}/\partial t$						${\partial T}_{skin,distal}/\partial t$					
	Female (7) (mean ± SD)		Male (7) (mean ± SD)		p-value (*MWU)*	CLES	Female (7) (mean ± SD)		Male (7) (mean ± SD)		p-value (*MWU)*	CLES
DC1	− 0.91	0.44	− 0.68	0.14	0.32	0.33	− 0.41	0.11	− 0.43	0.42	0.16	0.73
DC2	**− 0.45**	**0.07**	**− 0.34**	**0.04**	**0.01**	**0.08**	− 0.35	0.09	− 0.35	0.12	0.80	0.55
DC3	− 0.25	0.06	− 0.19	0.04	0.13	0.24	− 0.29	0.09	− 0.22	0.14	0.32	0.33
DC4	**− 0.16**	**0.04**	**− 0.22**	**0.05**	**0.05**	**0.82**	− 0.27	0.1	− 0.16	0.11	0.10	0.22
DC5	− 0.06	0.12	− 0.03	0.12	0.46	0.37	− 0.19	0.13	− 0.1	0.08	0.21	0.29
**DH1**	0.55	0.21	0.44	0.17	0.62	0.59	**0.04**	**0.13**	**0.21**	**0.15**	**0.05**	**0.18**
**DH2**	0.29	0.08	0.28	0.04	1.00	0.51	**− 0.01**	**0.13**	**0.27**	**0.13**	**0.01**	**0.10**
**DH3**	0.21	0.06	0.18	0.04	0.46	0.63	**0.08**	**0.16**	**0.29**	**0.14**	**0.02**	**0.12**
DH4	0.14	0.04	0.14	0.02	0.80	0.45	0.12	0.18	0.19	0.07	0.38	0.35
DH5	0.12	0.03	0.09	0.02	0.07	0.80	0.12	0.16	0.08	0.06	1.00	0.51
S	0.04	0.13	0.02	0.02	0.62	0.41	− 0.03	0.11	0.02	0.07	0.71	0.43

Significant values are in bold.

**Table 9:** Sex differences in thermal sensation vote and its relative change over the course of the last two partial-body cooling and warming transients.

	$TSV$						$(TSV-TSVi)/TSVi$					
	Female (7) (mean ± SD)		Male (7) (mean ± SD)		p-value (*MWU)*	CLES	Female (7) (mean ± SD)		Male (7) (mean ± SD)		p-value (*MWU)*	CLES
DC1	− 1.00	0.71	− 0.79	0.45	0.37	0.36	− 44.17	14.29	− 32.14	14.21	0.55	0.40
DC2	− 1.14	0.87	− 0.79	0.80	0.51	0.39	− 48.33	20.53	− 31.55	26.15	0.24	0.31
DC3	− 1.29	0.88	− 1.07	0.62	0.56	0.40	− 52.5	20.01	− 39.88	21.11	0.56	0.40
DC4	− 1.29	0.75	− 0.93	0.86	0.55	0.40	− 52.14	18.36	− 36.31	27.52	0.30	0.33
DC5	− 1.29	0.75	− 0.93	0.86	0.55	0.40	− 52.14	18.36	− 36.31	27.52	0.30	0.33
DH1	0.07	0.56	0.07	0.68	0.95	0.52	− 12.62	14.74	− 5.36	22.13	0.72	0.44
DH2	0.29	0.7	0.36	0.52	1.00	0.49	− 6.43	20.56	2.98	15.86	0.35	0.35
DH3	0.29	0.75	0.36	0.52	0.90	0.47	− 6.79	19.93	2.38	10.65	0.23	0.31
DH4	0.43	0.82	0.43	0.42	1.00	0.51	− 2.62	23.83	5.36	13.86	0.23	0.31
DH5	0.50	0.93	0.71	0.70	0.69	0.43	− 1.19	23.65	14.29	23.56	0.15	0.27
S	0.29	0.45	0.29	0.70	0.72	0.55	− 6.43	10.25	0.00	13.36	0.42	0.39

**Table 10:** Sex differences in thermal sensation vote and its relative change over the course of the last two partial-body cooling and warming transients.

	$TPV$					$(TPV-TPVi)/TPVi$						
	Female (7) (mean ± SD)		Male (7) (mean ± SD)		p-value (*MWU)*	CLES	Female (7) (mean ± SD)		Male (7) (mean ± SD)		p-value (*MWU)*	CLES
DC1	− 0.64	1.03	0.64	1.09	0.09	0.23	− 19.64	35.55	49.29	115.03	0.30	0.33
DC2	− 0.86	1.03	0.64	1.43	0.08	0.21	− 26.19	38.69	60.00	150.02	0.30	0.33
DC3	**− 1.36**	**0.83**	**0.64**	**1.43**	**0.04**	**0.16**	− 44.05	28.42	59.05	149.60	0.16	0.27
DC4	− 1.29	0.59	0.71	1.58	0.09	0.22	− 40.48	24.97	63.57	151.70	0.16	0.27
DC5	**− 1.57**	**0.56**	**0.71**	**1.48**	**0.02**	**0.12**	− 50.60	21.64	68.69	167.35	0.11	0.23
DH1	0.79	0.88	1.21	0.70	0.33	0.34	27.38	26.24	68.21	137.68	0.70	0.57
DH2	0.64	0.91	0.86	0.83	0.84	0.46	22.02	26.98	59.05	141.85	0.56	0.60
DH3	0.36	0.99	0.64	0.79	0.59	0.41	13.69	32.33	47.14	125.98	0.69	0.57
DH4	0.14	0.87	0.36	0.83	0.80	0.45	7.14	29.35	22.74	75.32	0.80	0.55
DH5	− 0.07	0.94	0.07	0.82	0.65	0.58	− 0.60	30.41	15.12	78.32	0.70	0.57
S	0.29	0.70	0.71	1.03	0.39	0.37	10.71	18.21	22.14	37.97	0.83	0.46

Significant values are in bold.

### Relationship between thermal perception and body temperatures

In the previous paragraphs, we have analysed differences in physiological and subjective data due to time-of-day and sex effects. We now want to understand whether the observed differences in thermal perception are only due to differences in skin temperature or whether time-of-day and sex also influence the relationship between skin temperature and thermal perception. A Mixed-effects Linear Model (MLM) with possible two-way interactions is employed to model the thermal sensation vote as a function of the skin temperature, the rate of change of skin temperature, sex, and time-of-day that are included as fixed effects. Given that $TSV$ does not depend linearly on ${T}_{skin,mean}$ and $\frac{{\partial T}_{skin, mean}}{\partial t}$ but rather reaches a positive and negative asymptote at $+3$ and $-3$ as we move away from the neutral skin temperature on the warm and cool side, we model this asymptotic behaviour with the hyperbolic tangent function ($Y=\mathit{tan}h\left(x\right)$) as already done in previous modelling works^77^. Thus, $\mathit{arctan}h\left(TSV/3\right)$ becomes our dependent variable, instead of $TSV$. Regression coefficients of the resulting linear model are shown in Table 11. The key assumptions of MLM (normality, homoscedasticity and no autocorrelation of the residual errors, no multicollinearity of the independent variables) have been checked and met. Sex was not found to affect the relationship between skin temperature and thermal sensation. However, we found that the time of the day influences the relationship between the skin temperature and its rate of change and the thermal sensation. In particular, at an equal rate of change in skin temperature, the participants felt cooler in the afternoon.

**Table 11:** Normal and standardized (Z-score) regression coefficients for the predictors of the thermal sensation vote in the MLM (727 observations and 14 groups).

	Coef	Standardized coef	Std.Err	z	p >|z|	[0.025	0.975]
$Intercept$	− 22.70	− 0.04	2.24	− 10.13	0.00	− 27.09	− 18.31
$Time$	10.05	0.10	2.83	3.55	0.00	4.50	15.61
${T}_{skin,mean}$	0.69	0.31	0.07	10.09	0.00	0.56	0.83
${T}_{skin,mean}$*$Time$	− 0.31	− 0.15	0.09	− 3.52	0.00	− 0.47	− 0.14
${\partial T}_{skin,mean}/\partial t$	2.18	0.19	0.17	12.84	0.00	1.85	2.51
${\partial T}_{skin,mean}/\partial t$*$\mathrm{Time}$	− 0.76	− 0.07	0.22	− 3.42	0.00	− 1.20	− 0.33
$Subject Var$	0.08	0.10	0.14				

### Thermal alliesthesia

**Figure 9:**
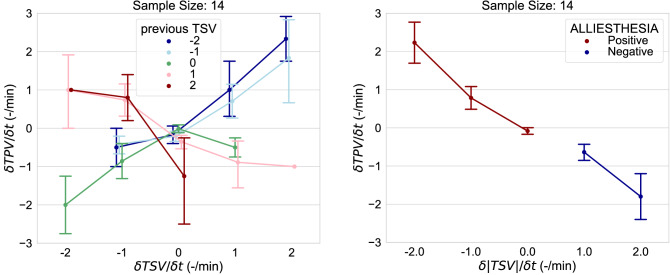
Rate of change of thermal pleasure as a function of the rate of change of thermal sensation (left) and rate of change of thermal pleasure as a function of the rate of change of the absolute value of the thermal sensation vote (right). Error bars represent one standard deviation.

Cabanac originally coined the term *“alliesthesia”* to refer to *“the property of a given stimulus to arouse pleasure or displeasure according to the internal state of the subject”*^78^. Any thermal stimulus that minimizes the *“thermoregulatory load error”* emanating from the body core is perceived as pleasant (*“positive alliesthesia”*), while any stimulus that exacerbates the discrepancy is perceived as unpleasant (*“negative alliesthesia”*)^78^. However, extant empirical evidence now shows that positive/negative alliesthesia can also be induced in the thermoneutral zone without any deviation in body core temperature^3–7,79–82^. This phenomenon is known as spatial thermal alliesthesia and occurs when the mean skin temperature is displaced from its *“neutral threshold”* and one or more body parts are heated or cooled to reduce/increase the whole-body discomfort. Here we show evidence that positive alliesthesial thermal states can also arouse during environmental transients when localized thermal discomfort is suddenly removed. This phenomenon can be referred to as temporal thermal alliesthesia. On the left of Fig. 9, we display the relationship between the rate of change of thermal sensation and thermal pleasure as a function of the previous thermal sensation vote. During the warming skin temperature transients, the warming thermal sensation overshoot ($$\frac{\partial TSV}{\partial t}>0$$) corresponded to an overshoot of thermal pleasure ($$\frac{\partial TPV}{\partial t}>0$$) but only when the participants previously felt cool ($$TSV<0)$$. Similarly, a cooling thermal sensation overshoot elicited positive alliesthesia overshoot only when the previous thermal sensation was on the warm side. To further analyse how the relationship between the thermal sensation and thermal pleasure overshoot is mediated by thermal alliesthesia, we derive a new metric which is the absolute rate of change of thermal sensation and is calculated as the rate of change of the absolute values of the thermal sensation ($$\frac{\partial \left|TSV\right|}{\partial t}=\frac{{\left|TSV\right|}_{t}{-\left|TSV\right|}_{t-1}}{\partial t}$$). This metric represents how fast the thermal sensation is counteracting the magnitude of the peripheral load error incurred in the antecedent exposure. A negative value implies that the thermal discomfort is decreasing (positive alliesthesia), while a positive value that is increasing (negative alliesthesia). On the right of Fig. 9 the rate of change of the thermal pleasure vote is plotted as a function of this new metric, and the instances of positive and negative alliesthesia can be clearly distinguished and a linear relationship emerges.

Finally, we employed MLM with possible two-way interactions to test whether $\frac{\partial TPV}{\partial t}$ depends on the previous thermal sensation vote, the current thermal sensation vote, the sex, and time-of-day other than the $\frac{\partial \left|TSV\right|}{\partial t}$. We could not find any statistically significant term other the $\frac{\partial \left|TSV\right|}{\partial t}$. This confirms that the intensity of thermal pleasure experienced in transitional environments depends on the corrective potential of the peripheral heat transfer; this information can help us to correctly model the temporal thermal alliesthesia phenomenon experienced during transitional thermal states and confirms what has been previously observed by Parkinson et al*.*^79^.

## Discussion

This study aimed to understand whether the time of the day affects human dynamic thermal perception. We have analysed thermal sensation votes collected after 120 min from the beginning of the afternoon and evening tests, *i.e.*, between 14:00 and 14:50 (afternoon) and 21:00 and 21:50 (evening) under sequences of partial-body active cooling and passive warming induced by a fan. We found that participants felt warmer during the evening warming transients and these results could not be explained in terms of differences in mean skin temperature which was the same in the afternoon and the evening. This was further confirmed by the fact that the relationship between mean skin temperature and the thermal sensation vote was not found to depend on the time of the day, with the same skin temperatures eliciting warmer sensory responses in the evening. However, we observed that the cutaneous vascular responses to the fan exposure were more pronounced in the evening. By analysing the times during which the experiments were conducted against the normalized mean course of the core body temperature as measured over several experiments^27,29,30,35^ and illustrated in Fig. 1, we can notice that our experiments were conducted during different body core temperature “*transition*” periods^83^: a “*heat gain*” phase in the afternoon (as the core body temperature climbs) and a “*heat loss*” phase in the evening (as the core body temperature starts to drop). Our findings suggest that humans are more sensitive to warming discomfort during the “*heat loss*” phase. This could be needed to favour the decrease of body core temperature during the cooling down phase. Similarly, the influence of light on thermophysiology has been mainly observed in the “*transition*” periods of the body core temperature^54^. Therefore, it seems particularly important to focus future thermal comfort studies on these periods. Furthermore, our results suggest that perceptual differences are only evident under dynamic conditions, while no differences are observed under steady-state conditions. This indicates that future investigations are to be aimed at the edges of the thermoneutral zone, as thermal sensory differences may be insignificant near the centre of neutrality.

In our study, we could also observe that females have a greater local vascular response at the neck than males. This corroborates previous evidence^69,84–88^, including our previous study^89^. The lower metabolic rate of females means they have lower metabolic heat-production capability during cold exposures and, thus, a poorer ability to thermoregulate compared to males which makes them more vulnerable to cool conditions. Hence, females must be more responsive to cooling transients through vasoconstriction to activate behavioural thermoregulatory responses earlier, which explains the observed higher cooling rates of the skin temperature at the neck.

## Limitations

In this study, the thermal adaptation time was equal to 70 min which is more than double the 30 min considered the norm in thermal comfort experiments. Despite this long adaptation time, skin temperatures up to 70 min from the start of the experiment were influenced by the previous thermal exposure in the office where the participants were required to stay for two hours to ensure that they were all having the same initial metabolic rate. As the main limitation of this study, we did not control the thermal conditions in the offices because we did not anticipate they could influence the results of the experiment. Because of this limitation and to eliminate any effect of the initial thermal state we only analysed time-of-day and sex effects on the physiological and subjective data collected 120 min from the start of the tests.

We did not measure core body temperature which would have allowed us to have more insights into the circadian phase of the participants.

Finally, the between-subject design and the small sample size of this study imply that there is a risk of making a Type II error and, thus, missing real effects (false negative results). This may have led to a failure in detecting differences in skin temperature between the afternoon and evening periods. A further limitation of the study related to the small sample size is that we could only focus on studying interindividual differences due to sex. Other than sex, individual differences can also be due to factors such as age, body build, fitness level, and geographic/ethnic acclimatization. The fact that the small sample is quite homogenous in terms of age and body build means that we could not study the impact of these factors and, thus, limits the generalisability of our results to the entire population.

## Conclusions

This study aimed to understand whether the time of the day (afternoon and evening) influences physiological (skin temperature) and subjective (thermal sensation and thermal pleasure) human thermal responses to a fan-induced dynamic localized thermal stimulus. It also investigated sex differences in human dynamic thermal perception. It was found that participants respond physiologically differently depending on the time of the day with a higher rate of change in the skin temperature observed in the evening compared with the afternoon. It was also observed that participants felt warmer in the evening during the warming skin temperature transients. This evidence suggests that humans are more sensitive to warming in the evening when the core body temperature starts to decline. This could be needed to favour the decrease of body core temperature. No differences were observed under steady-state thermal conditions. Furthermore, we found that time-of-day differences in thermal sensation could not be fully explained in terms of skin temperature, thus suggesting that equal skin temperatures elicit different sensory responses at different moments of the day. These results point to the importance of accounting for the time of the day when studying thermal comfort under dynamic conditions. Furthermore, given that the effect sizes observed were large, these results can contribute to developing novel building energy management algorithms for demand response. In particular, the shape of the set point temperature modulations could be optimized for thermal comfort and energy needs depending on the time of the day. The important practical implications of these findings highlight the need to conduct replication studies to confirm and extend these results and, therefore, facilitate applications in the built environment.

## Supplementary Information

Supplementary Table 1.

Supplementary Table 2.

Supplementary Table 3.

Supplementary Table 4.

Supplementary Information.

## Data Availability

The data can be accessed at https://doi.org/10.6084/m9.figshare.21878520.v1.
