# Supplementary material for: Effect of time-of-day on human dynamic thermal perception
Source: Sci Rep. 2023 Feb 9;13:2367. doi: 10.1038/s41598-023-29615-8 (PMC9911694; doi:10.1038/s41598-023-29615-8)
Supplement: Supplementary file 5 — Supplementary Information. [file 41598_2023_29615_MOESM5_ESM.docx]

The two thermal perception questions in English are reported below:

1. How do you feel right now?

◻ Cold ◻ Cool ◻ Slightly Cool ◻ Neutral

◻ Slightly Warm ◻ Warm ◻ Hot

2. Do you find it ...?

◻ Very Pleasant ◻ Pleasant ◻ Slightly Pleasant ◻ Indifferent

◻ Slightly Unpleasant ◻ Unpleasant ◻ Very Unpleasant

The two thermal perception questions translated into Italian are reported below:

1. In questo momento stai sentendo?

◻ Molto Freddo ◻ Freddo ◻ Leggero Freddo ◻ Né Caldo Né Freddo

◻ Leggero Caldo ◻ Caldo ◻ Molto Caldo

2. Lo trovi ...?

◻ Molto Piacevole ◻ Piacevole ◻ Leggermente Piacevole ◻ Indifferente

◻ Leggermente Sgradevole ◻ Sgradevole ◻ Molto Sgradevole

Supplementary Table 1. The measured horizontal spectral irradiance.

Supplementary Table 2. The measured vertical spectral irradiance.

Supplementary Table 3. Time-of-day differences throughout the test of the 5-minute resampled skin temperature at different locations.

Supplementary Table 4. Sex differences throughout the test of the 5-minute resampled skin temperature at different locations.

Supplementary Note. English version and Italian translation of the questionnaire.
